# Suitable Acidified Eggshell Powder Food Promotes *Brachionus calyciflorus* Growth and Reproduction: From Antioxidant Capacity Insight

**DOI:** 10.1155/2024/7875547

**Published:** 2024-05-31

**Authors:** Yang Danrong, Wang Li, Ma Xufa

**Affiliations:** College of Fisheries Huazhong Agricultural University, Wuhan 430070, China

## Abstract

Rotifers are natural initial bait for fish larvae in freshwater. Here, the effects of various concentrations of acidified eggshell powder solutions (0, 20, 40, 80, 160, and 320 mg/L) on the growth and reproduction of the rotifer *Brachionus calyciflorus* were evaluated in culture experiments (11 days). The population density, catalase (CAT) and superoxide dismutase (SOD) activity, and Na and Mg contents in rotifers were significantly higher in the 20–160 mg/L groups than in the control group. A redundancy analysis showed that the Na, Mg, Cr, K, and Ca contents were positively correlated with the rotifer population density and CAT and SOD activity. Furthermore, the generation time and lifespan of F2 rotifers were significantly higher in the 20–40 mg/L treatment groups (82.0 ± 3.7 hr and 162.0 ± 2.7 hr, respectively) than in the control group (64.0 ± 4.0 hr and 128.0 ± 4.0 hr, respectively). Average egg production in F2 rotifers was significantly higher in the 20–80 mg/L treatment groups (15.2 ± 0.7 individuals) than in the control group (11.7 ± 1.2 individuals). These results indicate that 20 mg/L eggshell powder is optimal for growth and reproduction in *B. calyciflorus*, providing a theoretical basis for using new mineral sources in high-quality open bait for fish larvae.

## 1. Introduction

Mineral element contents play an important role in biological processes [1]. In fish, mineral element deficiencies often result in a loss of appetite and low growth and survival rates [2]. Due to the low mineral content in natural water, fish must obtain supplemental minerals from feed (bait) to maintain normal activities. At present, egg yolk and powdered compound feed are the main baits for fish larvae in aquaculture [3]; however, nutrition and larval survival rates are insufficient.

Rotifers are natural opening baits for many fish larvae [4]. There is evidence that the growth rate of fish larvae is higher under feeding with *Brachionus calyciflorus* than with egg yolk [5] or pelleted feed [6]. However, for use as initial bait for fish larvae, it is necessary to feed rotifers a nutritional supplement, such as *Chlorella* [7]. *B. calyciflorus* fed only yeast exhibits a reduced unsaturated fatty acid content; fish larvae fed on this type of rotifer will die within a few days [8, 9]. Therefore, to meet the quantity and quality requirements for fish larvae opening bait, rotifers should be cultivated with food with high mineral element contents.

Chemical elements are involved in enzyme function and thereby could influence physical activity [10]. Superoxide dismutase (SOD) and catalase (CAT) are important enzymes in antioxidation and defense systems. Several studies have used SOD and CAT assays to evaluate antioxidant responses and mechanisms underlying the effects of test chemicals [11, 12]. For example, in response to various chemicals (pollutants), increased activity levels of SOD and CAT strengthen antioxidant responses to improve health [13]; alternatively, the inhibition of antioxidant responses can have negative effects on health. However, the effects of minerals on the population density, elemental composition, and antioxidant capacity in rotifers have not been determined.

Recent research has focused on the effects of single elements on rotifer reproductive rates, population density, and the hatching rate of dormant eggs [14, 15, 16, 17, 18]. Ca and Mg have synergistic effects on the physiological properties of rotifers [19]. When other conditions are suitable, 150 mg/L Ca^2+^ is optimal with respect to the rotifer reproductive rate; when the concentration exceeds 250 mg/L, the rotifer proliferation rate begins to decline [20]. Furthermore, the life span of rotifers is the longest when the concentration ratio of Ca^2+^ to Mg^2+^ is approximately 1 : 1.7 [20]. Zn and Al are essential trace metal elements. The addition of 0.4 mg/L Zn^2+^ can effectively promote the proliferation of rotifers. When the Zn^2+^ concentration in the culture environment is 0.0125–0.1000 mg/L over a long time period, the survival and fecundity of *B. rugosa* are adversely affected [18]. When the ZnCl_2_ concentration is greater than 1 mg/L, the maturation of *B. rugosa* is accelerated via a shortened lifespan and the inhibition of resting egg production [18]. When the concentration of Al^3+^ is 6 mg/L, the population density of a *Lecane* sp. is significantly reduced; however, at a concentration of 1.2 mg/L over a long culture time, the population density of rotifers decreases initially and then increases [16]. Therefore, it is of great significance to explore the optimal concentrations of minerals to promote the growth of rotifers.

Eggshells are currently used in agriculture as a mineral source [21]. Eggshells are mainly composed of 98% dry matter and 2% water [22]. They are rich in mineral elements, including Na, Ca, Mg, Ti, K, Cr, Pb, Fe, Zn, Ni, Cu, Mn, Al, As, Cd, and Sn [22]. In China, over 4 million tons of eggshells per year have been produced [23]. Owing to their availability, they should be considered in the production of rotifers for aquaculture. Though rotifer growth on algae is well-documented, additional strategies to improve rotifer nutritional quality are needed. In this study, we evaluated the effects of eggshell powder at various concentrations (i.e., 0, 20, 40, 80, 160, and 320 mg/L) on *B. calyciflorus*. In experiment I, we measured the population density, growth rate, element content, and enzyme activity of *B. calyciflorus*. In the monomer culture of experiment II, rotifer reproductive characteristics parameters were examined. We hypothesize that intermediate concentrations of eggshell powder are optimal for rotifer growth. This study provides a theoretical basis for the development of high-quality open bait for fish larvae.

## 2. Methods

### 2.1. Materials

#### 2.1.1. B. calyciflorus

*B. calyciflorus* is a commonly studied freshwater rotifer with a wide distribution. They are ideal for larviculture owing to their varied body sizes, high reproductive ability, and nutritional quality, which can be controlled with commercial enrichment products [8].

In this study, *B. calyciflorus* was collected and isolated from Qingnian Lake of Huazhong Agricultural University in June 2022. Rotifers were cultivated using deionized water after sterilization and aeration. Rotifers were fed *Chlorella vulgaris* at a density of 1 × 10^6^ cells/mL. *C. vulgaris* was obtained from the Freshwater Algae Culture Collection at the Institute of Hydrobiology, Wuhan, China, and cultivated in BG11 medium. Before exposure to experimental conditions, *B. calyciflorus* was cultured in a light incubator in the laboratory for over 2 months at the exponential growth phase.

#### 2.1.2. Eggshell Medium

Eggshells were purchased from Hong'an County, Hubei Province, in China. Before the experiment, the eggshells were cleaned, dried, crushed, and passed through a 400-mesh sieve. Then, the shell powder was dissolved in hydrochloric acid with a mass fraction of 40% to prepare a 10.0 g/L eggshell mother solution. The eggshell processing method was based in the methods described by Ajala et al. [22]. Different concentrations gradients of eggshell powder were made by adding deionized water to the eggshell mother solution according to the mother solution dilution method.

### 2.2. Experimental Design

When the eggshell powder concentration was above 400 mg/L in a preliminary experiment, the rotifers gradually died over 3 days. In this study, there were five concentrations of eggshell powder (20, 40, 80, 160, and 320 mg/L). There were six treatments, each replicated three times, including a control where *B. calyciflorus* was cultured in medium without eggshell powder (control). Both experiments were conducted in 50 mL Erlenmeyer flasks with 30 mL of deionized water (after sterilization and aeration for 24 hr) or eggshell medium (i.e., a mixture of deionized water and eggshell mother solution). The experiments were conducted under constant environments at 25 ± 1°C, 2,000 lx, and a light : dark period of 16 : 8 hr.

#### 2.2.1. Experiment I

To test the effect of eggshell powder on the population growth of *B. calyciflorus*, experiment I was designed as follows. At the beginning of the experiment, 30 individuals of *B. calyciflorus* were added to each replicate of each treatment group (i.e., each eggshell powder concentration). The rotifer culture density was 30 individuals per milliliter. During the experiment, the rotifers were incubated for 11 days, and the rotifer counts were recorded daily. At 9:00 every morning, 2 mL subsamples were taken from the Erlenmeyer flask using a 5 mL glass pipette and put in a centrifuge tube. This 2 mL subsample was fixed with 0.1 mL of 40% formaldehyde solution to obtain rotifer counts. After sampling, the treatment groups were supplemented with 2 mL of eggshell powder solution to maintain the corresponding concentration. After the end of the culture period, each experimental group was subjected to 24 hr of starvation in dark conditions for analyses of element contents and enzyme activity.

#### 2.2.2. Experiment II

Experiment II was designed to examine the effects of different concentrations of eggshell powder on the reproductive characteristics of *B. calyciflorus*. The experiment was conducted by placing 18 individuals of newly hatched rotifers (age: <2 hr) randomly caught from each Erlenmeyer flask into 24-well culture plates. Each well contained 1 mL of culture medium and one *B. calyciflorus* individual. Incubator culture conditions were the same as those in experiment I. Experimental individuals were transferred to fresh medium daily, and each rotifer and their offspring were monitored until death. During the initial 24 hr, the rotifers were checked every 2 hr, and the times of the first egg and neonate were recorded. Thereafter, the rotifers were checked every 12 hr, and the numbers of eggs and neonates produced as well as the number of original test individuals surviving were recorded.

### 2.3. Measurement of Population Growth and Reproductive Characteristics in Rotifers

#### 2.3.1. Population Density

Fixed rotifer samples were counted at 20x magnification using a microscope (Olympus SZ61). The counting results were converted into the number of rotifers per unit volume by the following formula:

(1) $$\begin{array}{c} N=\frac{n}{C}, \end{array}$$

where *N* is the number of individuals in 1 mL of culture system (individual per milliliter), *C* is the volume (mL), and *n* is the number of individuals obtained.

Based on the data collected, we derived various life history variables, including age-specific survivorship (*l*_*x*_) and reproduction (*m*_*x*_), net reproductive rate (*R*_0_), generation time (*T*), rate of population growth (*r*), life span (life expectancy, *e*_*o*_), and eggs/individual in each generation (i.e., F1 and F2) [24, 25].

#### 2.3.2. Generation Time

Generation time refers to the time from the birth of the parent to the birth of the larvae. It was calculated following Formulas (2) and (3) [24]:

(2) $$\begin{array}{c} R_{o}=\sum_{n=0}^{\infty }\left(l_{x}m_{x}\right), \end{array}$$

(3) $$\begin{array}{c} T=\frac{\sum_{n=0}^{\infty }\left(l_{x}m_{x}x\right)}{R_{o}}, \end{array}$$

#### 2.3.3. Rate of Population Growth

The rate of population growth (*r*) was calculated according to Formula (4) [24].

(4) $$\begin{array}{c} r=\frac{\ln R_{o}}{T}. \end{array}$$

#### 2.3.4. Life Span

Life span (life expectancy, *e*_*o*_) is the time from the birth of an individual to death [24]. Eggs per individual refers to the mean number of hatched eggs per rotifer.

### 2.4. Measurement of Element Contents and Enzyme Activity in Rotifers

#### 2.4.1. Mineral Element Content

Briefly, 10 mL of nitric acid was added to a digestion tube with 50 rotifers and maintained overnight. Then, the acid was heated and cooled to 25°C for an element content analysis. Elemental contents were measured using liquid-inductive coupling (ICP-OES) technology [26].

#### 2.4.2. Antioxidant Capacity

Briefly, 200 individuals of rotifers were placed into 1 mL of sterile water for an enzyme activity analysis. CAT activity and SOD activity levels were measured using the A007-1-1 and A001-1 kits, respectively (Nanjing Jiancheng Bioengineering Institute).

### 2.5. Statistical Analysis

The effects of eggshell powder at various concentrations on the rotifer density, mineral element contents, enzyme activity, and life table parameters were analyzed using one-way analysis of variance (ANOVA). Multiple comparison tests were used to compare sample means (LSD and SNK). The significance level was set at *p* < 0.05. The principal component analysis (PCA) was used to evaluate variation in element contents. Redundancy analysis (RDA) was carried out to explore the relationships between rotifer density, mineral element contents, and antioxidant capacity. The density, CAT activity, and SOD activity of *B. calyciflorus* were selected as response variables, and the contents of Na, Ca, Mg, Al, K, Si, Fe, Cd, Cu, and Cr in rotifers were selected as explanatory variables. All statistical processing and analyses were carried out using SPSS ver. 22 and Origin Pro 2022.

## 3. Results

### 3.1. Species Density and Growth Rates

The population density of rotifers in the control and treatment groups showed a continuous increase with the prolongation of culture time. However, on the 10th day, the density of rotifers decreased in the treatment groups with concentrations of 40–80 mg/L (Table 1). The effect of eggshell powder on rotifer density was time-dependent. On the 3rd day of the experiment, there was a significant difference (*p* < 0.05) in rotifer density between the 40 mg/L group and the control group. On the 7th day, there was a significant difference in rotifer density between the 40–160 mg/L eggshell powder treatment groups and the control group (*p* < 0.05). On the first 10 days of the experiment, the densities of rotifers in the 20–160 mg/L groups were higher than that in the control group. Afterward, the rotifer density in the 320 mg/L treatment group was higher than that in the control group (Table 1).

**Figure 1:**
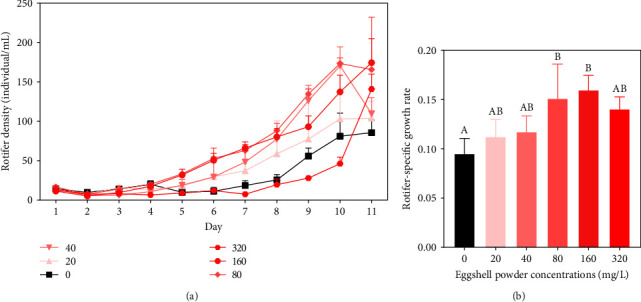
Rotifer densities after treatment with various eggshell powder concentrations. (a) Effects of various eggshell powder concentrations on rotifer-specific growth rates. (b) Values are presented as the mean ± SE. Bars that do not share the same superscript letters are significantly different (*p* < 0.05).

The specific growth rates of rotifers in the 160–320 mg/L treatment groups were significantly higher than that of the control group (*p* < 0.05), with increases of 34.64%–49.4% (Figure 1).

### 3.2. Mineral Element Content and Antioxidant Capacity

**Figure 2:**
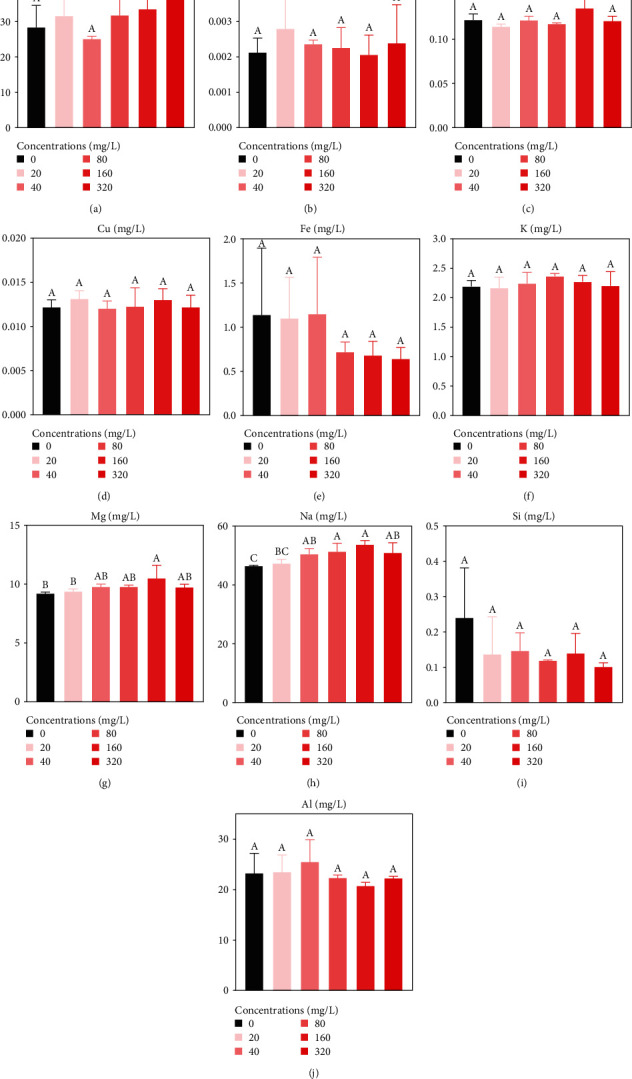
Effects of eggshell powder on the contents of 10 mineral elements after 10 days of treatment. (a) Ca; (b) Cd; (c) Cr; (d) Cu; (e) Fe; (f) K; (g) Mg; (h) Na; (i) Si; and (j) Al. Values are presented as the mean ± SE. Bars that do not share the same superscript letters are significantly different (*p* < 0.05).

Contents of 10 elements in *B. calyciflorus* were evaluated. The changes in the average content of each element across all treatment groups, from most to least, were as follows: Na (50.8071 ± 4.4970 mg/L) > Ca (35.2801 ± 13.5952 mg/L) > Al (25.2919 ± 5.2436 mg/L) > Mg (10.4461 ± 1.3315 mg/L) > K (2.1699 ± 0.2461 mg/L) > Fe (1.2534 ± 0.7597 mg/L) > Si (0.2256 ± 0.1750 mg/L) > Cr (0.1436 ± 0.0349 mg/L) > Cu (0.0126 ± 0.0021 mg/L) > Cd (0.0029 ± 0.0017 mg/L) (Figures 2(a), 2(b), 2(c), 2(d), 2(e), 2(f), 2(g), 2(h), 2(i), and 2(j)). There were significant differences in the Na and Mg contents of *B. calyciflorus* between the treatment and control groups (*p* < 0.05) (Figures 2(g) and 2(h)). The Na content increased significantly by 108.66%–115.60% in the 40–320 mg/L treatment group (*p* < 0.05). The Mg content displayed increased significantly by 113.98% in the 160 mg/L treatment group (*p* < 0.05).

**Figure 3:**
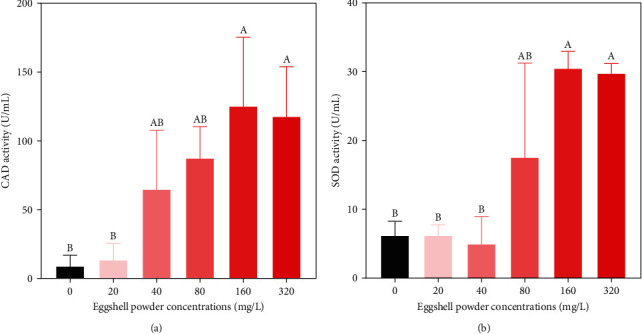
Effects of eggshell powder on the antioxidant capacity of CAD (a) and SOD (b) after 10 days. Bars that do not share the same superscript letters are significantly different (*p* < 0.05). CAT, catalase; SOD, superoxide dismutase.

The rotifer CAT activity in the experimental group was significantly higher than that in the control group. For an increasing eggshell powder concentration, CAT activity increased initially and then decreased. However, there was no significant difference in CAT activity between the 20–80 eggshell mg/L treatment group and the control group (*p* > 0.05). These results revealed that although the addition of eggshell powder can activate CAT enzyme activity, it does not influence CAT enzyme activity within the range of 20–80 mg/L. For concentrations of 160 mg/L and 320 mg/L, CAT activity increased significantly by 13.83 and 13.00 times over that in the control group (*p* < 0.05) (Figure 3(a)).

As the eggshell powder concentration increased, SOD activity showed an increasing trend, followed by a slight decrease at 320 mg/L. SOD activity did not differ significantly between the treatment groups at 20–80 mg/L and the control group (*p* > 0.05), indicating that a low concentration of eggshell powder does not significantly affect the activation of SOD in rotifers. SOD activity in the 160 mg/L and 320 mg/L treatment groups was 4.90 and 4.78 times higher, respectively, than that in the control group (*p* < 0.05) (Figure 3(b)).

### 3.3. Relationships between the Rotifer Density, Mineral Element Contents, and Antioxidant Capacity

**Figure 4:**
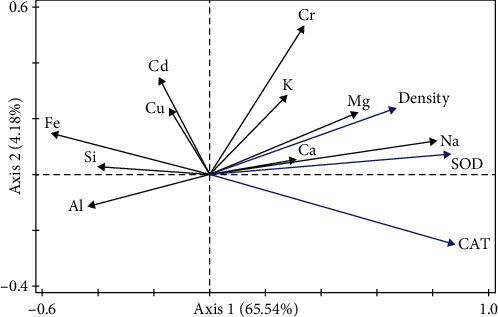
Redundancy analysis of the effects of 10 mineral element contents on the rotifer density, SOD, and CAT after 11 days. CAT, catalase; SOD, superoxide dismutase.

As shown in Table 2, the three most important elements were Na (60.30%), Fe (11.90%), and Ca (9.50%), with a total contribution rate of 81.70%. The RDA revealed that rotifer density is positively correlated with Na > Mg > Cr > K > Ca and negatively correlated with Fe > Al > Si > Cu > Cd. In the RDA of CAT activity in rotifers, positive correlations were observed for Na > Mg > Ca > K > Cr, and negative correlations were observed for Fe > Al > Si > Cd > Cu. In an RDA of SOD activity in rotifers, positive correlations were observed for Na > Mg > Cr > Ca > K, and negative correlations were observed for Fe > Al > Si > Cd > Cu. These results revealed that Na, Mg, and Fe were the most important elements contributing to the changes in rotifer density and antioxidant capacity. Moreover, rotifer growth was positively related to Na and Mg and was negatively related to the Fe content (Figure 4).

### 3.4. Average Egg Production, Generation Time, and Life Span

**Figure 5:**
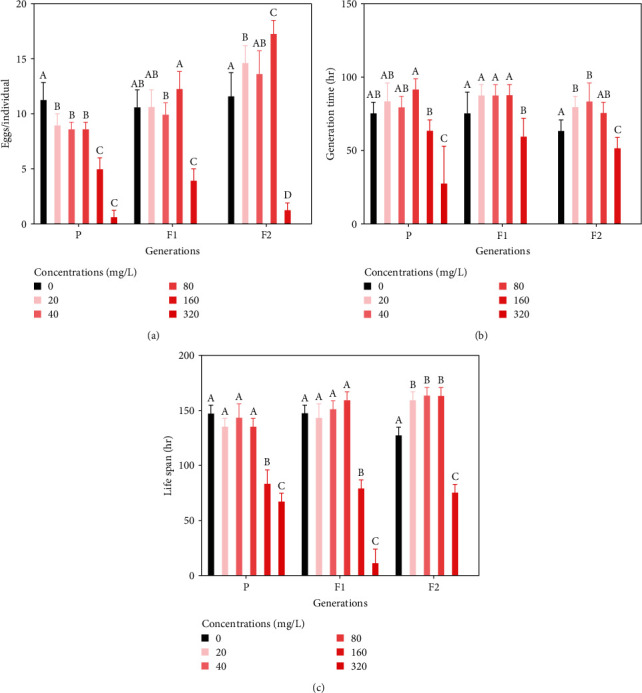
Effects of eggshell powder on the average egg production (a), generation time (b), and life span (c). Values are presented as the mean ± SE. Bars that do not share the same superscript letters are significantly different (*p* < 0.05).

The effects of eggshell powder on life table parameters for parents (P) and first (F1) and second (F2) generation offspring are shown in Figure 5. The average egg production estimates for P, F1, and F2 in the 160–320 mg/L treatment groups were significantly lower than that in the control group (*p* < 0.05). In particular, in the treatment group with a concentration of 320 mg/L, the average egg production for F1 and F2 was zero. The average egg production rates for F2 in the 20–80 mg/L treatment groups were higher than that in the control group and increased by 17.14%–48.57%. The generation times for P, F1, and F2 in the 160–320 mg/L treatment groups decreased significantly (*p* < 0.05). The generation time for F2 individuals at 2–40 mg/L was significantly higher than that in the control group (*p* < 0.05). In the 160–320 mg/L treatment groups, the life spans of P, F1, and F2 decreased significantly; however, the life span of F2 at 40–80 mg/L was significantly longer than that in the control group (*p* < 0.05).

## 4. Discussion

### 4.1. Effects of Eggshell Powder on Population Dynamics

We detected slightly higher rotifer population growth in the 20–160 mg/L treatment groups than in the control group. These findings indicated that the addition of low concentrations of eggshell powder could promote rotifer proliferation. There are several explanations for these results. First, the surface of eggshell powder has several 0.4-*μ*m-diameter cylindrical pores, which can absorb harmful substances, such as metabolic waste products [27]. Second, eggshell powder contains a variety of mineral elements, which can affect the metabolism of rotifers. In this study, the Mg and Na contents were significantly positively correlated with rotifer density. Previous studies have also shown that the proper concentrations of Ca^2+^ and Mg^2+^ play an important role in the proliferation of rotifers [19, 28, 29]. For example, the addition of 150 mg/L Ca^2+^ can promote the rapid reproduction of rotifers; however, if the Ca^2+^ concentration exceeds 250 mg/L, the proliferation rate of rotifers slows down according to the theory of calcium senescence [29]. Mg^2+^ is essential for biological activities, such as nutrient absorption, antioxidation, and antimetabolism. Ca^2+^ and Mg^2+^ interact with each other. When the concentration ratio of Ca^2+^ and Mg^2+^ is approximately 1 : 1.7, the lifespan of rotifers is the longest [20].

Although the density of rotifers in the 40 mg/L concentration group quickly reached the peak value on day 10, the population density dropped sharply on day 11. It is possible that the rotifer population could not be stably maintained due to spatial competition among excessive rotifer individuals in a specific culture system. Alternatively, a chemical substance released by the rotifers themselves could cause a rapid decline in rotifer density [30]. Alternatively, the metabolic waste from rotifers may exceed the adsorption capacity of eggshell powder in the 10-day cultivation system, which decreases rotifer density [31]. In addition, rotifer density showed a decrease during the first 3 days during the experiment period. It is possible that low-density inoculation (50 individuals/L) requires larger culture systems; otherwise, there will be a temporary decrease in rotifer density [32]. In this study, both experiments were conducted in a 50 mL culture system.

To further evaluate the influences of eggshell powder on population dynamics, a monomer culture was carried out to explore the reproductive characteristics of rotifers. The average egg number per rotifer individual can be influenced by the spawning interval and reproductive period [14, 33]. The average egg number per rotifer was positively correlated with the spawning interval for the same reproductive period. In this study, the average egg number per rotifer showed a significant difference from that in the control when the concentration was 80–320 mg/L. This may be related to a shortened reproductive period and the prolongation of the spawning interval. In a previous study, when other conditions were suitable, there was a significant decrease in rotifer population growth under high salinity conditions [34]. This is consistent with the results of this study showing that the generation time of rotifers is significantly shorter in the 160–320 mg/L treatment groups. The average egg number per F1 and F2 individual showed an increasing trend in the 20–40 mg/L treatment groups. It is speculated that under the low salinity conditions, the energy required to regulate the osmotic pressure of aquatic organisms decreases, thereby increasing egg production [35].

### 4.2. Effect of Eggshell Powder on Antioxidant Capacity and Mineral Elements

In addition to the increases in rotifer density and egg production, with an increasing eggshell powder concentration, the activity levels of SOD and CAT and the contents of mineral elements such as Ca^2+^ and Cu^2+^ also increased. There were no statistically significant differences in SOD and CAT enzyme activities between the control group and the 20–80 mg/L eggshell powder treatment groups. However, the SOD and CAT enzyme activities in the 160–320 mg/L treatment groups were significantly higher than that in the control group. This result is consistent with those of previous studies showing that SOD and CAT activities increase significantly under high salinity conditions [34]. Expression levels of antioxidant-related genes were also significantly altered in response to salinity changes in *Brachionus plicatilis* [35]. The increased activity of SOD and CAT might be related to the extension of the rotifer lifespan [36]. The RDA showed that Ca^2+^ was the most important explanatory factor for antioxidant enzyme activity, followed by Na^+^ and Mg^2+^. Mineral elements might have an important impact on the activity of antioxidant enzymes. A previous study has shown that Cu^2+^ protects blue crabs (*Callinectes sapidu*s) from oxidative damage via increases in SOD and CAT activity [37]. Ca^2+^ can significantly increase the activity of antioxidant enzymes to protect against adverse environmental conditions [38]. Caloric restriction results in reactive oxygen species production and increases the activity of MnSOD significantly in *B. plicat*a [39]. Adding the appropriate quantity of Mg^2+^ can promote the activity of SOD [40]. High Mg^2+^ concentrations have an inhibitory effect on CAT enzyme activity [41]. *B. calyciflorus* adapts to a high-Na^+^ salinity water environment by increasing antioxidant enzyme activity [18].

Overall, our results suggested that the rotifer lifespan is closely related to mineral element contents via changes in SOD and CAT activity. A high concentration (exceeding 160 mg/L) of eggshell powder triggered the activation of antioxidant enzymes to adapt to adverse circumstances [42], whereas a low concentration of eggshell powder (20 mg/L) is conducive to the growth and reproduction of *B. calyciflorus*. Nevertheless, we acknowledge the potential for species-specific effects of eggshell powder and the need for further research to optimize its use for different rotifer species. Additionally, future laboratory experiments should compare the effects of rotifers (grown on eggshell compounds) and algae alone as larval fish food. These analyses can provide guidance for the development of aquatic feeds.

## 5. Conclusions

Our results indicate that a high concentration of eggshell powder increases the activities of antioxidant enzymes in rotifers. A low concentration of eggshell powder increases the generation time, egg production, and lifespan of rotifers. Therefore, it is inferred that 20 mg/L eggshell powder is conducive to the growth and reproduction of *B. calyciflorus*. Moreover, eggshell powder had an important impact on offspring fitness. Eggshell powder has a low cost and is widely availability, and this study provides a theoretical basis for its use in the development of a high-quality open bait for fish larvae.

## Figures and Tables

**Table 1:** Rotifer densities after treatment with various concentrations of eggshell powder at different time points.

Time (days)	Concentrations (mg/L)					
	0	20	40	80	160	320
1	14.17 ± 3.75	14.67 ± 3.06	12.83 ± 1.04	13.67 ± 2.47	15.00 ± 2.18	14.83 ± 2.47
2	9.83 ± 3.06	9.33 ± 3.01	9.83 ± 2.36 ^*∗*^	8.00 ± 1.00	7.00 ± 1.00 ^*∗*^	6.00 ± 1.00
3	14.33 ± 4.62	15.17 ± 5.67	15.67 ± 7.378 ^*∗∗*^	15.83 ± 1.89	11.67 ± 2.36	9.33 ± 2.60 ^*∗*^
4	19.17 ± 5.69	20.17 ± 4.63 ^*∗*^	17.17 ± 3.37 ^*∗∗*^	13.67 ± 1.89	12.33 ± 2.36	12.00 ± 2.60 ^*∗∗*^
5	10.00 ± 5.29	10.17 ± 5.58	11.17 ± 5.25	18.17 ± 8.13 ^*∗∗*^	20.83 ± 8.61 ^*∗∗*^	20.83 ± 8.62
6	11.33 ± 4.91	12.00 ± 6.06	15.67 ± 6.21	29.67 ± 18.04 ^*∗∗*^	27.33 ± 20.41 ^*∗∗*^	26.67 ± 20.82
7	18.67 ± 6.01	16.17 ± 3.33	19.00 ± 2.00 ^*∗*^	37.33 ± 31.82 ^*∗∗*^	47.67 ± 26.50 ^*∗∗*^	51.50 ± 20.97
8	25.83 ± 6.81	28.33 ± 11.07	30.17 ± 10.30 ^*∗∗*^	58.83 ± 41.71 ^*∗∗*^	75.17 ± 40.82 ^*∗∗*^	87.67 ± 20.10
9	55.83 ± 10.30	44.83 ± 8.13	45.83 ± 9.75 ^*∗*^	77.50 ± 64.53 ^*∗∗*^	110.00 ± 61.83	131.67 ± 24.83
10	81.17 ± 23.95	71.67 ± 17.23	69.50 ± 55.94 ^*∗∗*^	103.00 ± 65.61 ^*∗∗*^	133.83 ± 60.91	165.83 ± 19.92
11	85.83 ± 15.78	102.67 ± 26.53	97.33 ± 27.71 ^*∗*^	104.00 ± 21.55 ^*∗∗*^	105.00 ± 23.25	106.17 ± 22.03

*Note*:  ^*∗*^*p*  < 0.05 and  ^*∗∗*^*p* < 0.01, for the comparison between treatment and control groups under the same incubation time.

**Table 2:** Summary of principal component analysis and redundancy analysis of elements after 11 days.

Elements	Variance explained (%)	Contribution (%)	Pseudo-*F*	*p*-Value
Cu	0.80	1.10	0.30	0.824
K	0.60	0.80	0.20	0.910
Al	1.20	1.60	0.30	0.794
Mg	0.90	1.20	0.30	0.712
Fe	8.60	11.90	2.70	0.096
Cr	2.40	3.30	0.90	0.324
Na	43.90	60.30	12.50	0.002
Cd	4.10	5.60	1.30	0.254
Ca	6.90	9.50	2.50	0.200
Si	3.40	4.70	1.20	0.290

## Data Availability

The data that support the findings of this study are available from the corresponding author upon reasonable request.
